# Activin A Secreted by Cancer-Associated Fibroblasts Reduces the Sensitivity of Breast Cancer Cells to Ixazomib via Inhibition of Proteasome Activity

**DOI:** 10.3390/biom15091318

**Published:** 2025-09-15

**Authors:** Shuaiming Geng, Siyao Liu, He Liu, Siao Wang, Yichen Niu, Jing Gao, Yong Meng, Mingqing Gao

**Affiliations:** 1 School of Medicine, Northwest University, Xi’an 710069, China; 202233088@stumail.nwu.edu.cn (S.G.); 202324253@stumail.nwu.edu.cn (S.W.); 202434415@stumail.nwu.edu.cn (Y.N.); 202422182@stumail.nwu.edu.cn (J.G.); 2 College of Life Sciences, Northwest University, Xi’an 710069, China; 202332664@stumail.nwu.edu.cn (S.L.); 202310284@stumail.nwu.edu.cn (H.L.); 3 Department of Oncology Surgery, Xi’an No. 3 Hospital—The Affiliated Hospital of Northwest University, Xi’an 710018, China

**Keywords:** breast cancer, cancer-associated fibroblasts, drug resistance, INHBA/Activin A, ixazomib

## Abstract

Breast cancer (BC) remains a leading cause of cancer-related mortality among women globally, and the role of cancer-associated fibroblasts (CAFs) in promoting BC progression is well established. Ixazomib, a proteasome inhibitor approved for the treatment of multiple myeloma, has demonstrated therapeutic potential in BC in preclinical trials. However, whether its efficacy is influenced by the tumor microenvironment, particularly CAFs, remains unclear. This study aims to investigate the role of CAFs with high expression of Activin A (encoded by INHBA) in modulating the sensitivity of BC cells to ixazomib. We demonstrate that ixazomib exhibited significant cytotoxicity in BC cells, but high-INHBA CAFs compromise ixazomib cytotoxicity through ERK-mediated proteasome suppression, reversible by Activin A antagonism. Additionally, the overexpression of INHBA in fibroblasts reduces the efficacy of ixazomib in xenograft models. Clinical data analysis revealed that high INHBA expression is associated with poor prognosis in BC patients and reduced immune cell infiltration. These findings suggest that targeting INHBA in CAFs could enhance the therapeutic efficacy of ixazomib in BC, particularly in patients with low INHBA expression. This study provides novel insights into the role of CAFs in drug resistance and identifies INHBA as a potential therapeutic target.

## 1. Introduction

Breast cancer is one of the most common cancers in women and remains the leading cause of cancer death in women worldwide [1]. Ixazomib, a second-generation proteasome inhibitor, has been approved by the FDA for the treatment to multiple myeloma. Ixazomib inhibits proteasome activity by preferentially binding to the β5 subunit site of the 20 S proteasome, leading to an imbalance in intracellular proteostasis that triggers apoptosis [2,3,4]. Recent studies show that ixazomib also has anti-tumor effects on solid tumors such as breast cancer [5]. Clinical trials have showed that ixazomib, as a proteasome inhibitor, shows great potential in breast cancer treatment and can significantly improve the prognosis of breast cancer patients [6,7,8]. However, it has not been approved by the FDA to treatment patients with breast cancer. In view of this, in-depth study of the role of ixazomib in breast cancer treatment is important for optimizing the therapeutic effect, improving the survival rate of patients and expanding the scope of the application of ixazomib.

Cancer-associated fibroblasts (CAFs) are the major stromal cells in the tumor microenvironment and play important roles in the development of tumors, including breast cancer [9,10]. Increasing evidence suggests that CAFs can promote tumor growth, invasion, angiogenesis and immunosuppression, and are potential therapeutic targets for cancer [11]. Moreover, CAFs are involved in the development of tumor drug resistance [12,13]. It is generally accepted that CAFs promote tumor development and drug resistance, but different fibroblast subsets play different roles in cancer progression [14]. Despite considerable advances, the understanding of CAFs reducing chemosensitivity is still limited. We wonder whether ixazomib could be used to treat breast cancer patients, and whether CAFs affect the sensitivity of breast cancer cells to ixazomib, which is a critical step in expanding the therapeutic applications of ixazomib.

INHBA, as a member of the TGF-β superfamily, encodes the βA subunit of inhibin, which can form Activin A through disulphide bonds [15]. Evidence suggests that Activin A can regulate the malignant phenotype of tumor cells through signaling pathways such as SMAD, PI3K-AKT, and ERK [16], and follistatin (FST) is its specific antagonist [17]. Recent studies have found that Activin A derived from the tumor microenvironment can induce drug resistance in HER2-positive breast cancer [18]. However, whether Activin A affects the sensitivity of breast cancer cells to the proteasome inhibitor ixazomib remains unclear.

In this study, we demonstrated whether ixazomib has significant cytotoxic effects on breast cancer cells and CAFs through in vitro and in vivo experiments, whether CAFs mediate this process, and the possible mechanisms.

## 2. Materials and Methods

### 2.1. Isolation and Culture of CAFs

CAFs were isolated from fresh breast tumor tissues collected after surgical removal at the Affiliated Hospital of Northwest University. This was approved by the Medical Ethics Committee of Northwestern University and consented to by the patients. Briefly, tumor tissue samples were rinsed with phosphate-buffer solution (PBS) and cut into tissue pieces of less than 1 mm^3^. Then, the pieces were digested in DMEM/F12 (Gibco, Grand Island, NY, USA) containing 1 mg/mL collagenase (Sigma, Shanghai, China), 0.25 mg/mL hyaluronidase (Sigma), and 10% fetal bovine serum (FBS) for 2 h at 37 °C. Subsequently, digested tissues were filtered through a cell sieve, and the supernatant was discarded by centrifugation. Finally, the cell pellets were suspended in DMEM/F12 containing 10% FBS and cultured in an incubator at 37 °C with 5% CO_2_.

### 2.2. Cell Lines and Culture

Breast cancer cell lines MCF7 and MDA-MB-231, 293T cells, and human BJ fibroblasts were purchased from the National Collection of Authenticated Cell Cultures (Shanghai, China). All cells used in this study were negative for mycoplasma and were cultured in DMEM/F12 containing 10% FBS at 37 °C with 5% CO_2_.

### 2.3. RNA-Sequence (RNA-Seq) and Data Process

Total RNA was extracted from CAFs using TransZol Up reagent (TransGen, Beijing, China), and Nano Drop ND-1000 was used to control the amount and purity of the total RNA. Reverse transcription was performed to obtain cDNA and construct specific libraries. The library products were sequenced using Illumina NovaseqTM 6000 (LC-Bio Technology Co., Ltd., Hangzhou, China) for bipartite sequencing. We used the following process for RNA-seq data analysis: raw data filtering and quality control were first performed using Cutadapt-1.9, and data quality was verified by FastQC-0.11.9. Subsequently, sequencing reads were compared to the human reference genome using HISAT2. Based on the comparison results, we applied StringTie-2.1.6 and Ballgown-2.40.0 software for transcript quantification and expressed mRNA expression levels as FPKM values. Differential analysis was performed by DESeq2, and the screening criteria were genes with FDR < 0.05 and fold change ≥ 2 [19]. Finally, KEGG pathway analysis, GO functional annotation, and GSEA enrichment analysis were performed on the differentially expressed genes.

### 2.4. Preparation of CAF-Conditioned Medium (CM)

CAFs were cultured in DMEM/F12 containing 10% FBS to approximately 70% confluence and washed twice with PBS. Fresh culture medium was added and incubated for 24 h in an incubator with 5% CO_2_ at 37 °C. The medium was collected and used as CAF-CM after centrifugation.

### 2.5. Cell Counting Kit-8 (CCK-8) Assay

Cells were cultured into 96-well plates at 2 × 10^3^/well and incubated in DMEM/F12 containing 10% FBS for 24 h. Then, after treatment with ixazomib (Selleck, Houston, TX, USA), Activin A (MCE, Monmouth Junction, NJ, USA), or its antagonist FST (MCE), the old medium was replaced by fresh medium containing 10% CCK8 solution (TransGen), following 2 h of incubation at 37 °C. Finally, the absorbance of the wells at 450 nm was measured using a microplate reader (BioTek Epoch2 Instruments, Agilent, Santa Clara, CA, USA).

### 2.6. Western Blotting

Total cellular proteins were extracted using RIPA lysate (Beyotime, Shanghai, China) and protein concentrations were determined by a BCA protein assay kit (Solarbio, Beijing, China). Equal amounts of proteins were separated by SDS-PAGE and transferred to polyvinylidene difluoride membranes, followed by blockage with 10% skim milk to block non-specific binding sites. The membranes were incubated with antibodies against p-AKT (Abclonal, Wuhan, China), p-ERK1/2 (Abclonal), AKT (Abclonal), ERK1/2 (Abclonal), or GAPDH (Proteintech, Wuhan, China). This followed incubation with corresponding peroxidase-labeled secondary antibodies goat anti-mouse (Proteintech) or goat anti-rabbit (Proteintech). Protein bands were visualized using a chemiluminescence kit (Thermo, Waltham, MA, USA) and were acquired by an imaging system (LAS-4000, Fujifilm, Tokyo, Japan)

### 2.7. Real-Time Quantitative PCR (RT-qPCR)

Total RNA was extracted from cells using the TransZol Up reagent (TransGen). RNA concentration was measured using a microplate reader, and all samples were adjusted to a uniform concentration of 500 ng/μL. Reverse transcription was then performed by using the PrimeScript^TM^ RT Master Mix kit (Takara Bio, Kyoto, Japan) to synthesize cDNA. In the RT-qPCR experiment, GAPDH was selected as the internal reference gene, and amplification was performed using PerfectStart^®^ Green qPCR SuperMix (TransGen) and specific primers. The expression difference of the target gene was ultimately calculated using the 2^(−ΔΔCt)^ relative quantification method [20]. The primer sequences for INHBA were as follows: Forward Primer, GGAGCTCAGACAGCTCTTACC; Reverse Primer, ATCTCCGAGGTCTGCTCCAT. GAPDH: Forward Primer, GGAGTCCACTGGCGTCTTCA; Reverse Primer, GTCATGAGTCCTTCCACGATACC.

### 2.8. ELISA

ELISA was performed to determine the secretion level of Activin A in different cells. Briefly, cells were cultured in DMEM/F12 containing 10% FBS until about 70% confluence; then, the cells were washed with PBS and cultured in fresh medium for another 24 h. Subsequently, the culture medium was collected and Activin A in medium were measured using the Activin A ELISA kit (Huzhen, Shanghai, China) assay according to the manufacturer’s instructions.

### 2.9. Determination of Proteasome Activity

We employed a proteasome activity assay kit (Abcam, Cambridge, MA, USA) to measure chymotrypsin-like activity in this study. This assay is based on 7-Amino-4-methylcoumarin (AMC) fluorescently labeled peptide substrates, which release free, highly fluorescent AMC in the presence of proteolytic activity. The fluorescence intensity is detected to quantitatively analyze proteasome activity. The specific operational steps were performed according to the manufacturer’s instructions with minor modifications.

Briefly, cells were collected and lysed using NP-40 cell lysis buffer, which contained 50 mM Tris-HCl (pH 7.4), 150 mM NaCl, and 1% NP-40. This was supplemented with sodium pyrophosphate, β-glycerophosphate, sodium orthovanadate, sodium fluoride, EDTA, and leupeptin as protease/phosphatase inhibitors.

For each sample, we set up a pair of sample reaction wells. Then, 25 µL sample and 1 µL proteasome inhibitor were added into one well, and 25 µL sample without proteasome inhibitor was added into another well. The total volume of both wells was adjusted to 100 µL with assay buffer. Then, 1 µL proteasome substrate was added into each sample well and incubate for 20 min (T_1_) at 37 °C to warm up the reaction mix, protected from light; this was followed by another 30 min (T_2_) incubation at 37 °C. The fluorescence output at time points T_1_ and T_2_ was, respectively, measured using a fluorometric microplate reader (SynergyH1, Agilent) at Ex/Em = 350/440 nm. The proteasome activity was presented as relative fluorescence unit (RFU), defined as the amount of fluorescent products produced from the substrate under the action of the proteasome per minute at 37 °C. The formula is RFU = [(tFU_2_ − iFU_2_) − (tFU_1_ − iFU_1_)]/(T_2_ − T_1_), where tFU is the measured value of the sample which does not contain proteasome inhibitor but shows total proteolytic activity, iFU is the value of the sample containing proteasome inhibitor but which shows non-proteasome activity, and the number is the time point.

### 2.10. Transfection with Lentiviral Vector

The INHBA gene sequence was cloned into the TK-PCDH-copGFP-T2A-Puro lentiviral vector. We mixed the plasmid (INHBA:pMD2.G:psPAX2 = 2 μg:1 μg:2 μg) and Lipofectamine 3000 with a fresh serum-free, antibiotic-free culture medium and added it dropwise to 293T cells for virus packaging; then, the viral solution was concentrated, purified, and used to infect the BJ fibroblasts. Finally, the increased expression level of INHBA in transfected BJ cell (INHBA-BJ) was validated by RT-qPCR and ELISA after screening with puromycin (Beyotime). BJ cells transfected with empty vectors (Vector-BJ) were used as control.

### 2.11. Tumor-Bearing Mice

Female nude mice (BALB/cNj-Foxn1^nu^/Gpt, Huachuang Sino, Taizhou, China) were housed in pathogen-free facilities with individual ventilation, constant humidity (40–60%) and temperature (23 ± 1 °C), free feeding and drinking, and regular 12 h light/12 h dark cycles. At age of 6 weeks, mice were randomly divided into three groups of six mice each. MCF7 cells (6 × 10^6^) and INHBA-BJ (3 × 10^6^) cells or Vector-BJ (3 × 10^6^) were co-injected into the right flanks of mice, and mice that were only injected with MCF7 (6 × 10^6^) cells were used as controls. Tumor volume was monitored every 4 days after tumor formation at the injection sites, and ixazomib was administered intraperitoneally at a drug concentration of 7 mg/kg once a week after the average tumor volume reached the size of 100 mm^3^. All animal programs were approved by the Experimental Animal Management and Ethics Committee of Northwestern University.

### 2.12. Analyses Based on Public Datasets

INHBA expression levels in tumor tissues of patients with breast cancer were processed using the GEPIA (http://gepia2.cancer-pku.cn/#index, accessed on 10 August 2024) tool for TCGA and GTEx database. The clinical prognostic value of INHBA expression in breast cancer patients was assessed using the Kaplan–Meier Plotter (https://kmplot.com, accessed on 16 August 2024) for evaluation. The correlation between INHBA and immune infiltration was analyzed by TIMER 2.0 (http://timer.cistrome.org/, accessed on 28 August 2024).

### 2.13. Statistical Analysis

All data are expressed as the mean ± SEM of three independent experiments. Statistical analyses were performed using GraphPad Prism v.8 and comparisons were performed using two-sided paired Student’s *t*-test or one-way ANOVA. Significant differences were considered as follows: ns, non-significant, * *p* < 0.05, ** *p* < 0.01.

## 3. Results

### 3.1. CAFs Reduced the Sensitivity of MCF7 Cancer Cells to Ixazomib

**Figure 1:**
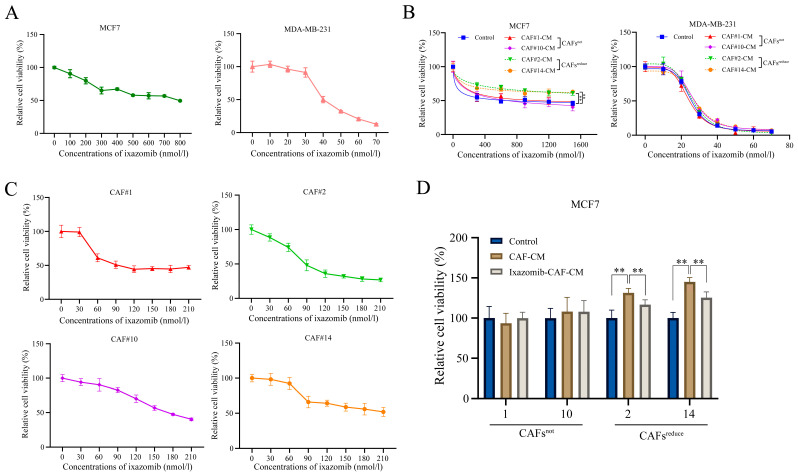
CAFs reduced the sensitivity of MCF7 cancer cells to ixazomib. (**A**) Relative cell viability curves for MCF7 or MDA-MB-231 cells under ixazomib treatments with a range of concentrations. *n* = 4. (**B**) Inhibitory effects of ixazomib with increasing concentrations on MCF7 or MDA-MB-231 relative cell viability (%). Cells were cultured in regular medium (Control) or CAFs-CM. 1#, 2#, 10# and 14# represent different individual patients. *n* = 4, ** *p* < 0.01 by one-way ANOVA. (**C**) Inhibitory effects of ixazomib on CAFs^not^ and CAFs^reduce^ viability. *n* = 6, results are expressed as relative cell viability (%). (**D**) Effect of ixazomib treatment at IC50, calculated based on (**A**), on MCF7 cell viability when cells were cultured in regular medium, CAF-CM or ixazomib-pretreated CAF-CM. *n* = 4, ** *p* < 0.01 by one-way ANOVA, results are expressed as relative cell viability (%).

We found that ixazomib had a significant dose-dependent toxic effect on both MCF7 and MDA-MB-231 breast cancer cells at indicated concentration ranges when they were cultured in regular complete medium (Control) (Figure 1A). However, when cancer cells were cultured in conditioned medium (CM) of CAFs extracted from different individuals, arbitrarily named CAF1#, CAF2#, CAF10# and CAF14#, CAF2#-CM and CAF14#-CM, they reduced the sensitivity of MCF7 cancer cells to ixazomib, while CAF1#-CM and CAF10#-CM did not induce similar changes compared with the control (Figure 1B, left). By contrast, CM of CAFs extracted from each individual did not change the sensitivity of MDA-MB-231 cells to ixazomib (Figure 1B, right). This result suggests that CAFs from each individual may have unique characteristics and functions, and they have different effects on different types of breast cancer cells. In addition, ixazomib also showed a dose-dependent toxic effect on CAFs from all 4 individuals within the concentration range from 0 to 210 nmol/L (Figure 1C).

Based on the different effects of these CAFs from different patients on the sensitivity of MCF7 cancer cells to ixazomib, we divided CAFs from different patients into two groups, one of which was designated as CAFs^reduce^, meaning CAFs that reduce the sensitivity of MCF7 cells to ixazomib (CAF2#, CAF14#), while the other was named CAFs^not^, meaning CAFs that have no effect on the sensitivity of MCF7 cells to ixazomib (CAF1#, CAF10#). Notably, CAFs only affected the sensitivity of MCF7, but not MDA-MB-231 breast cancer cells, to ixazomib, a phenomenon that prompted us to select MCF7 cells as a model for subsequent mechanistic studies in order to deeply resolve the mechanism of CAF-mediated resistance.

Finally, ixazomib at the concentration of IC_50_ was used to treat MCF7 cells that were, respectively, cultured in regular complete medium, CAF-CM, and ixazomib-pretreated CAF-CM. For CAFs^not^, both CAF-CM and ixazomib-pretreated CAF-CM did not affect the sensitivity of MCF7 cells to ixazomib as indicated by cell viabilities compared with the control. For CAFs^reduce^, cell viabilities in CAF-CM groups were higher than those in both control and ixazomib-pretreated CAF CM groups, suggesting CAF-CM could reduce the sensitivity of MCF7 cells to ixazomib, and this inductive effect was weakened if CAFs^reduce^ groups were pretreated by ixazomib (Figure 1D).

These results collectively demonstrate that CAFs^reduce^ is the primary factor responsible to reduce breast cancer cell sensitivity to ixazomib. Notably, ixazomib exhibits synergistic pharmacotoxicity against both CAFs and cancer cells, suggesting its potential therapeutic efficacy in some breast cancer patients.

### 3.2. Screening CAFs^reduce^-Secreted Crucial Proteins That Contribute the Resistance of MCF7 Cells to Ixazomib

**Figure 2:**
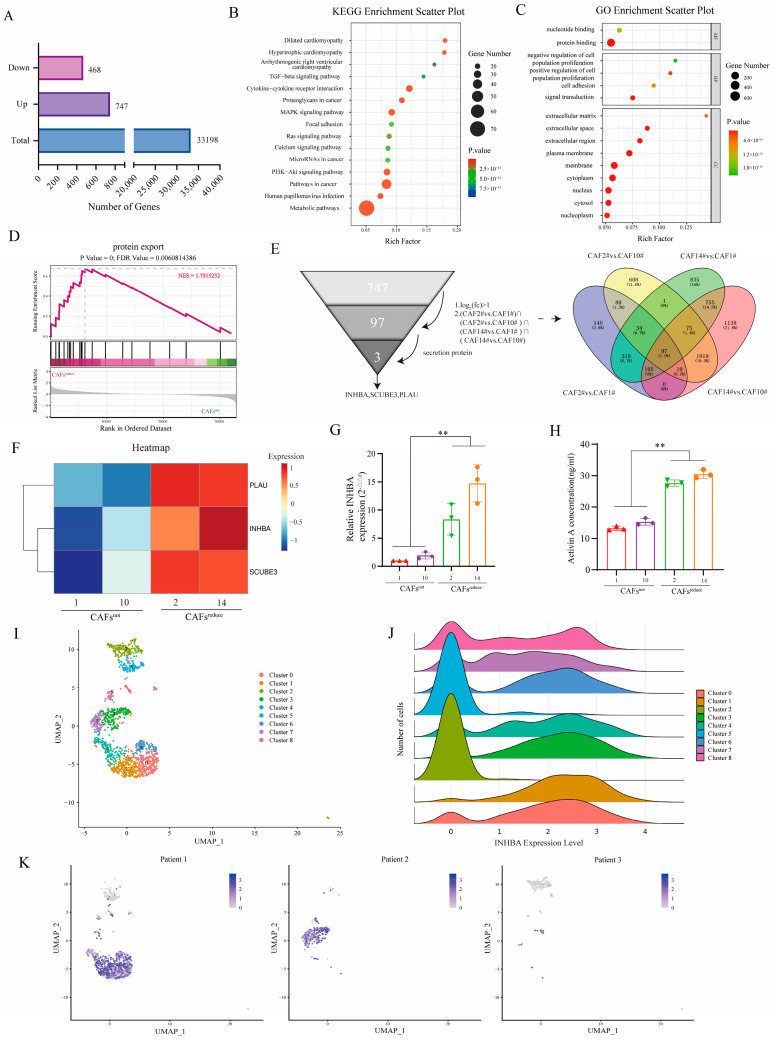
The screening of crucial secretory proteins for CAFs^reduce^ to contribute the resistance of MCF7 cells to ixazomib. (**A**) A histogram showing the number of total and DEGs for CAFs^not^ compared to CAFs^reduce^. (**B**,**C**) Top 15 Kyoto Encyclopedia of Genes and Genomes (KEGG) and Gene Ontology (GO) enrichment analyses of DEGs. (**D**) GSEA of protein export signaling pathway between CAFs^not^ and CAFs^reduce^. (**E**) The screening process of candidate DEGs between CAFs^not^ and CAFs^reduce^ that caused differences in ixazomib sensitivity of cancer cells (**left**). The intersection of four groups of differentially up-regulated genes ((CAF2# vs. CAF1#) ∩ (CAF2# vs. CAF10#) ∩ (CAF14# vs. CAF1#) ∩ (CAF14# vs. CAF10#)) (**Right**). (**F**) A heatmap of average expression trends of PLAU, INHBA and SCUBE3 genes in CAFs^not^ and CAFs^reduce^. (**G**) INHBA expression levels in CAFs^not^ and CAFs^reduce^ were determined by RT-qPCR analysis. *n* = 3, ** *p* < 0.01 by Student’s *t*-test. (**H**) The protein secretion levels of Activin A in CAFs^not^ and CAFs^reduce^ were measured by ELISA. *n* = 3, ** *p* < 0.01 by Student’s *t*-test. (**I**) The UMAP plot of single-cell RNA sequencing (scRNA-Seq) data displaying CAF subclusters of breast tumor tissues, with color by sample type. (**J**) Aridge plot of INHBA expression in CAF subclusters based on scRNA-Seq data, with color by sample type. (**K**) Differences in INHBA expression in CAFs derived from each patient, with color coding (gray to blue) for expression of INHBA.

RNA-Seq was performed to investigate the molecular characteristics of CAFs^reduce^ and CAFs^not^. A total of 33,198 co-expressed genes were detected between CAFs^reduce^ and CAFs^not^, and differential gene expression (DEG) analysis showed that 747 genes were up-regulated and 468 genes were down-regulated in CAFs^reduce^ compared to CAFs^not^ (Figure 2A).

In order to reveal the potential functions of these DEGs, we performed Kyoto Encyclopedia of Genes and Genomes (KEGG) and Gene Ontology (GO) enrichment analyses. The signaling pathways associated with cancer progression in the KEGG enrichment analysis results mainly included TGF-β pathway, MAPK pathway, PI3K-Akt pathway, cytokine–cytokine receptor interaction, focal adhesion, and chemokine signaling, etc. (Figure 2B). GO analysis showed that DEGs were significantly enriched for 15 functional terms: 2 terms were enriched from the molecular function ontology, 4 terms were enriched from the biological process ontology, and 9 terms were enriched from the cellular component ontology. The pathways related to cancer crosstalk mainly included cell adhesion, extracellular matrix (ECM), extracellular space, extracellular region, etc. (Figure 2C). We further performed gene set enrichment analysis (GSEA) on the transcriptomic data and observed a significant enrichment of protein output signals in CAFs^reduce^ (Figure 2D).

Of the 747 up-regulated genes in CAFs^reduce^, 97 genes were overexpressed in CAFs^reduce^ These showed more than a 1-fold change compared to CAFs^not^ and were common to the set of four differentially up-regulated genes ((CAF2# vs. CAF1#) ∩ (CAF2# vs. CAF10#) ∩ (CAF14# vs. CAF1#) ∩ (CAF14# vs. CAF10#)). Since CAF-CM reduced the sensitivity of MCF7 to ixazomib, we preferred to select those differentially up-regulated genes that encoded secretory proteins for the subsequent analysis among the 97 differentially up-regulated genes. After filtering, three secretory protein-encoding genes—PLAU, inhibin beta A (INHBA), and SCUBE3—were screened as candidates (Figure 2E), and they had higher expression levels in CAFs^reduce^ than in CAFs^not^ (Figure 2F).

Among these three genes, the up-regulation of INHBA genes has been reported to be a key factor in the development of breast cancer [21,22], and it plays important roles in drug resistance in many types of tumors [18,23]. Thus, we finally selected INHBA as a target gene in this study. We further verified the mRNA expression and protein secretion levels of INHBA in the two groups of CAFs by RT-qPCR and ELISA, respectively. Results showed that the expression level of the INHBA gene (Figure 2G) and the secretion of its encoding protein, Activin A, (Figure 2H), were significantly higher in CAFs^reduce^ compared to CAFs^not^.

We further reanalyzed the data of single-cell sequencing obtained from tumor tissues of 3 breast cancer patients in our previous work [24]. A total of 9 CAF clusters were identified based on marker genes (Figure 2I). These CAF subpopulations showed significant differences in INHBA expression: high expression was presented in clusters 0, 1, 3, 4, 6, 7, and 8, whereas the expression levels were lower in clusters 2 and 5 (Figure 2J). Notably, the CAF subcluster with high INHBA expression was predominantly enriched in samples from patients 1 and 2, while expression was almost undetectable in samples from patient 3 (Figure 2K). We subsequently analyzed the GSE176078 single-cell RNA-seq dataset. The results confirmed that the expression of INHBA was significantly cell-type-specific and mainly restricted to the CAF population (Supplementary Figure S1). More importantly, we observed significant heterogeneity in the expression level of INHBA in CAFs among different patients, with some patients having significantly higher expression than others (Supplementary Figure S2).

These results suggested that CAFs from different breast cancer patients expressed different mRNA levels of INHBA. INHBA might be a crucial functional factor in CAFs, derived from different individuals, contributing the resistance of MCF7 cells to ixazomib.

### 3.3. Blockade of CAFs-Secreted Activin a Restores the Sensitivity of MCF7 Cells to Ixazomib

Given that CAFs^reduce^ secreted higher levels of Activin A than CAFs^not^, we speculated that Activin A mediated CAFs induced a decrease in the sensitivity of MCF7 cells to ixazomib.

**Figure 3:**
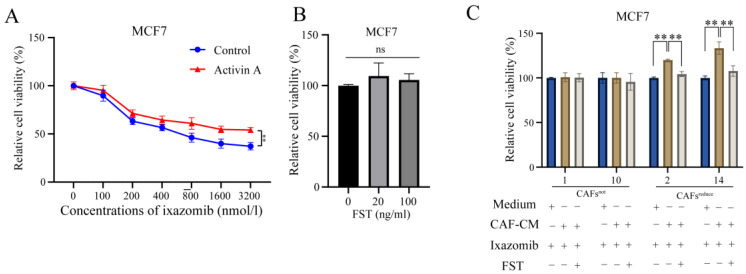
The blockade of CAF-secreted Activin A restores the sensitivity of MCF7 cells to ixazomib. (**A**) Comparison of effects of ixazomib on MCF7 cell viability when it was cultured in medium with or without recombinant Activin A. *n* = 5, ** *p* < 0.01 vs. control by one-way ANOVA. (**B**) Effects of FST on MCF7 relative cell viability (%). *n* = 4, ns, non-significant by Student’s *t*-test. (**C**) Effect of ixazomib treatment on MCF7 relative cell viability, when cells were cultured in regular medium, CAF-CM, or CAF-CM+FST. *n* = 3, ** *p* < 0.01 by one-way ANOVA. Regular medium: medium.

To evaluate this, we used ixazomib to treat MCF7 cells that were cultured in complete medium with or without exogenous recombinant Activin A. Results showed that Activin A attenuated the sensitivity of MCF7 cells to ixazomib as indicated by relative cell viability (Figure 3A).

We next investigated whether FST, an antagonist of Activin A, [17] could reverse the sensitivity decrease of MCF7 cells to ixazomib, induced by CAFs^reduce^. First, we screened appropriate concentrations (20 and 100 ng/mL) at which FST did not directly affect the cell viabilities (Figure 3B) and could only function as an antagonist of Activin A. Further, we observed that ixazomib has a significant toxic effect on MCF7 compared to control, and the toxic effect was weakened when cells were cultured in CAF^reduce^-CM, indicating the protective effect of CAFs^reduce^ from ixazomib-caused toxicity. Importantly, this protective effect of CAFs^reduce^ was attenuated when Activin A in CAFs^reduce^-CM was neutralized by FST. However, CAFs^not^ did not show similar protective effects to CAFs^reduce^. (Figure 3C). These data suggest that Activin A secreted by CAFs^reduce^ reduces the sensitivity of MCF7 cells to ixazomib.

### 3.4. CAFs^reduce^-Secreted Activin a Reduces the Sensitivity of MCF7 Cells to Ixazomib Through Inhibiting Proteasome Activity

**Figure 4:**
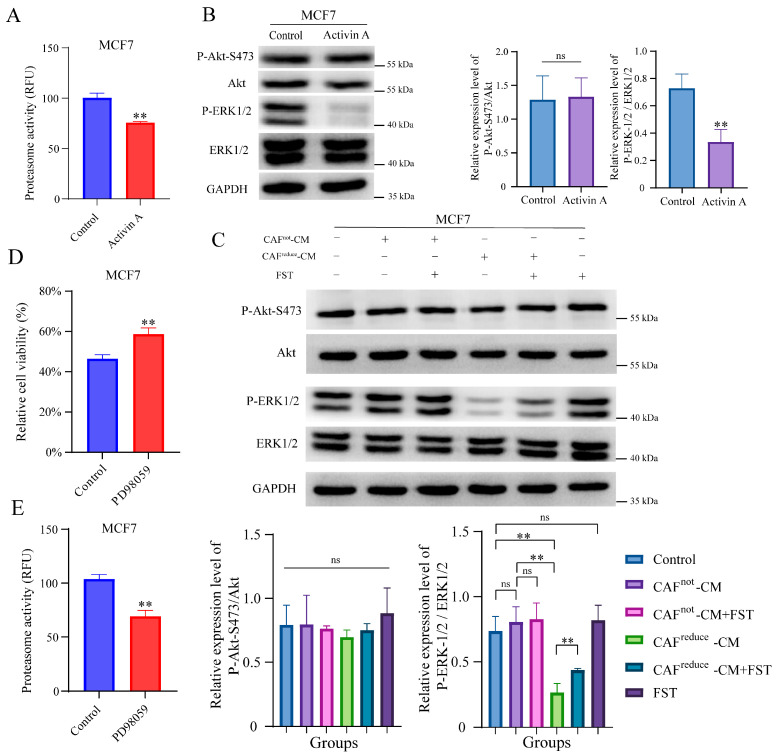
CAF^reduce^-secreted Activin A reduces the sensitivity of MCF7 cells to ixazomib by inhibiting proteasome activity. (**A**) Relative proteasome activity of MCF7 measured by proteasome activity ELISA kits. Cells were cultured in medium with or without Activin A. *n* = 3; ** *p* < 0.01 by Student’s *t*-test. (**B**) MCF7 cells were cultured in medium with or without Activin A, and the protein expression of p-ERK1/2, ERK1/2, p-AKT, and AKT was detected by Western blotting (original images can be found in Figure S3). *n* = 3, ns, non-significant, ** *p* < 0.01 by Student’s *t*-test. (**C**) MCF7 cells were cultured in normal medium, CAF^not^-CM (with or without FST), and CAF^reduce^-CM (with or without FST), respectively, and the protein expression of p-ERK1/2, ERK1/2, p-AKT, and AKT in the cells was detected by Western blotting (original images can be found in Figure S3). *n* = 3, ns, non-significant, ** *p* < 0.01 by one-way ANOVA. (**D**) Comparison of relative cell viability (%) of ixazomib on MCF7 when they were cultured in medium with or without ERK inhibitor (PD98059). *n* = 5, ** *p* < 0.01 by Student’s *t*-test. (**E**) Relative proteasome activity of MCF7 measured by proteasome activity ELISA kits. Cells were cultured in medium with or without ERK inhibitor (PPD98059). *n* = 3, ** *p* < 0.01 by Student’s *t*-test.

It is reported that when cancer cells have low proteasome activity, they face low proteotoxic pressure. Thus, less cells died when treated with proteasome inhibitors [25,26]. Ixazomib is an oral proteasome inhibitor that has been approved for the treatment of multiple myeloma [27]. To investigate whether CAFs^reduce^-secreted Activin A reduces the sensitivity of MCF7 cells to ixazomib through inhibiting proteasome activity, the recombinant Activin A was used to treat MCF7 cells. As we expected, Activin A treatment did inhibit proteasome activity of MCF7 cells (Figure 4A).

Previous studies suggested that Activin A exert biological functions by regulating MAPK/ERK and PI3K/AKT signaling pathways [28,29]. Here, Western blotting results showed that treatment with recombinant Activin A significantly inhibited the phosphorylation level of ERK in MCF7 cells, but it did not affect the phosphorylation level of AKT (Figure 4B).

Furthermore, we observed that the phosphorylation level of ERK in MCF7 cells cultured in CAFs^reduce^-CM was also inhibited compared to those cells cultured in CAFs^not^-CM, and this inhibition effect was attenuated by the antagonist of Activin A, FST, indicating that the higher level of Activin A secreted by CAFs^reduce^ inhibited the phosphorylation of ERK in MCF7 cells, and FST did not affect the phosphorylation level of ERK in MCF7 cells cultured in both the regular medium and CAFs^not^-CM. In addition, FST did not affect the activation of AKT in MCF7 cells at any culture conditions (Figure 4C).

We also assessed whether the inhibition of the ERK signal pathway resulted in reduced the sensitivity of MCF7 to ixazomib. We found PD98059, an inhibitor of ERK pathway, could reduce the sensitivity of MCF7 cells to ixazomib (Figure 4D). Interestingly, PD98059 also inhibited the proteasome activity of MCF7 cells (Figure 4E).

In summary, Activin A secreted by CAFs reduced the sensitivity of MCF7 cells to ixazomib, which may be related to the inhibition of proteasome activity mediated by the ERK1/2 signaling pathway.

### 3.5. Up-Regulation of INHBA in CAFs Reduces the Sensitivity of MCF7 to Ixazomib In Vivo and In Vitro

**Figure 5:**
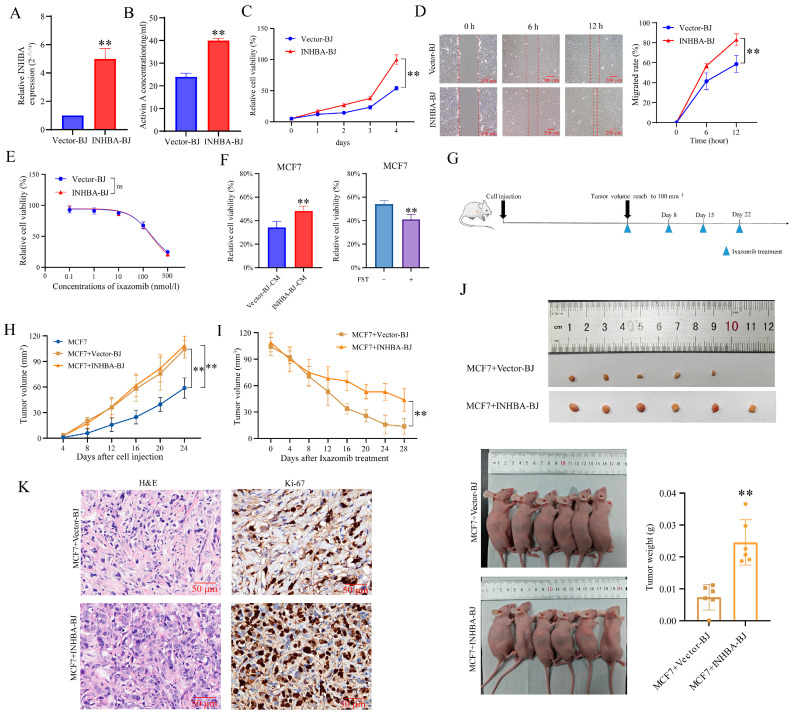
Up-regulation of INHBA in CAFs reduces ixazomib sensitivity of MCF7 in vivo and in vitro. (**A**) INHBA expression levels in Vector-BJ and INHBA-BJ determined by RT-qPCR analysis. *n* = 3, ** *p* < 0.01 by Student’s *t*-test. (**B**) Protein secretion levels of INHBA in Vector-BJ and INHBA-BJ measured by ELISA. *n* = 3, ** *p* < 0.01 by Student’s *t*-test. (**C**) Effect of INHBA overexpression on BJ relative cell viability assayed by CCK-8 kit. *n* = 6, ** *p* < 0.01 by Student’s *t*-test. (**D**) Effect of INHBA overexpression on BJ cell migration evaluated by scratch assay. *n* = 4, ** *p* < 0.01 by Student’s *t*-test. (**E**) Effect of INHBA overexpression on relative cell viability of BJ cell induced by ixazomib. *n* = 4, ns, non-significant. (**F**) CCK-8 assay evaluating effect of ixazomib on MCF7 cell viability. MCF7 cells were cultured in conditioned medium from BJ cells overexpressing Activin A (INHBA-BJ-CM) or vector control medium (Vector-BJ-CM) (**Left**); or INHBA-BJ-CM either treated with neutralizing antibody FST (neutralization group) or untreated (non-neutralized group) (**Right**), to assess ixazomib’s effect on MCF7 cell activity under different conditions. *n* = 4, ** *p* < 0.01 by Student’s *t*-test. (**G**) A flow chart of animal experiments. Ixazomib was administered intraperitoneal at drug concentration of 7 mg/kg once weekly after average tumor volume reached size of 100 mm^3^. (**H**) Tumor growth curves after tumor formation at cell injection sites of nude mice before tumor volume reaches to 100 mm^3^. *n* = 6. ** *p* < 0.01 by Student’s *t*-test. (**I**) Curves of tumor volume changes in nude mice within 28 days after 1st administration of ixazomib. *n* = 6. ** *p* < 0.01 by Student’s *t*-test. (**J**) Photographs of extracted tumor and mice (**left**) and statistical analysis of mice tumor weights (**right**). *n* = 6, ** *p* < 0.01 by Student’s *t*-test. (**K**) Representative images of H&E and Ki-67 staining of xenograft tissue sections.

To further validate the above functions of INHBA genes, we constructed a cell line (INHBA-BJ) that stably overexpressed INHBA in both mRNA expression (Figure 5A) and protein secretion level (Figure 5B). We found that INHBA overexpression promoted BJ fibroblast viability (Figure 5C) and migration (Figure 5D), but did not alter its own sensitivity to ixazomib (Figure 5E).

When MCF7 cells were in vitro cultured in CM of INHBA-BJ, their sensitivity to ixazomib was reduced compared to those cells cultured in CM of Vector-BJ, as relative cell viability. Importantly, this protective effect of INHBA-BJ was also attenuated when Activin A in INHBA-BJ-CM was neutralized by FST (Figure 5F).

Subsequently, we evaluated whether INHBA-BJ induced the in vivo resistance of MCF7 cells to ixazomib by using a subcutaneous co-injection model (Figure 5G). Results showed that the average volume of tumors formed at injection sites of mice in both two co-injection groups reached 100 mm^3^ at 24 days after injection, and it was significantly larger than that in mice that received MCF7 cell injection alone at indicated time points, but no difference was observed between MCF7+Vector-BJ and MCF7+INHBA-BJ groups (Figure 5H). The results demonstrate that fibroblasts promote tumor growth, while INHBA overexpression in BJ fibroblasts showed no significant effect on tumor proliferation capacity compared with the MCF7+Vector-BJ group.

Within 28 days after ixazomib treatment, the average tumor volume gradually decreased in two co-injection groups, but the reduction in tumor volume in INHBA-BJ group was significantly smaller than that in MCF7+Vector-BJ group (Figure 5I). In addition, the extracted tumor weight of mice in MCF7+INHBA-BJ group was higher than that in the MCF7+Vector-BJ group at the end of the experiment (at 28 days after ixazomib treatment) (Figure 5J). The pathological features of the xenografts were confirmed by the HE staining of tissue sections; a further immunohistochemically assay using Ki-67 revealed a marked increase in cellular proliferation in the MCF7+INHBA-BJ group compared to the MCF7+Vector-BJ controls (Figure 5K). The data from this study indicate that CAFs overexpressing INHBA primarily induce drug resistance in MCF7 cells through an Activin A-mediated mechanism, unlike CAFs with low expression of INHBA.

### 3.6. Analysis of the Clinical Relevance of INHBA Expression with Breast Cancer

**Figure 6:**
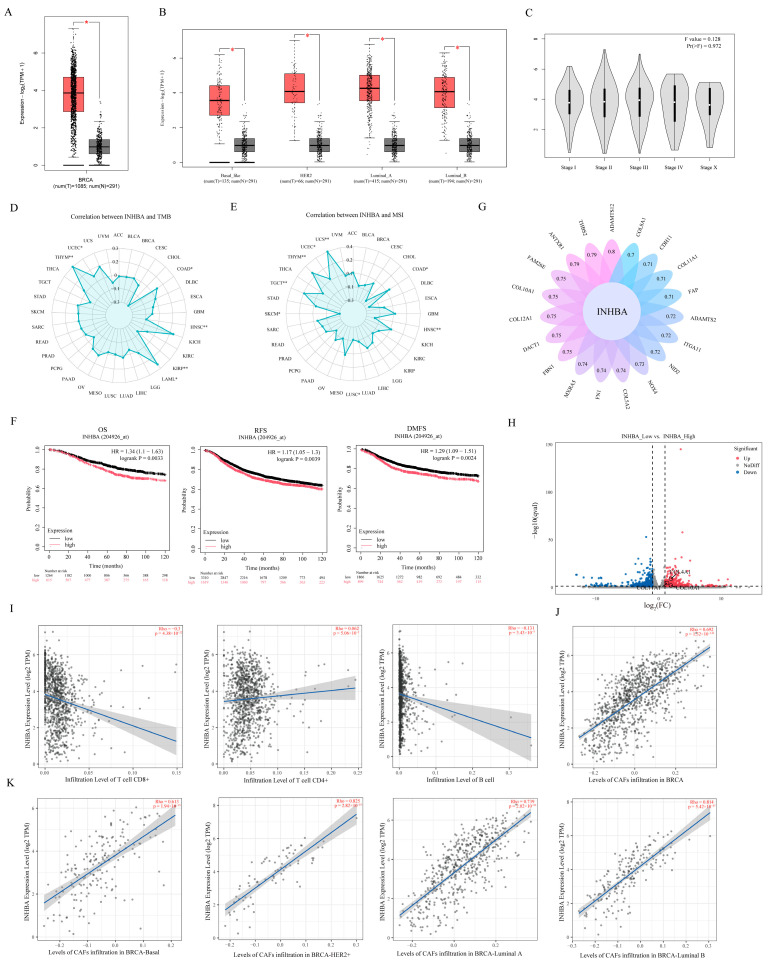
Analysis of clinical relevance of INHBA expression with breast cancer. (**A**,**B**) INHBA expression levels in BRCA (*n* = 1085), basal-like (*n* = 135), HER2+ (*n* = 66), Luminal A (*n* = 415), Luminal B (*n* = 194) and normal tissues (*n* = 291) analyzed in TCGA database. * *p* < 0.05. BRCA: breast cancer. (**C**–**E**) Correlation analysis between INHBA expression levels and breast cancer stage (**C**), tumor mutational load (TMB) (**D**) and microsatellite instability (MSI) (**E**) based on TCGA database. (**F**) Kaplan–Meier survival plot comparison between patients with low and high expression of INHBA based on TCGA database. Split patients by upper tertile. OS: overall survival (*n* = 1879), RFS: recurrence-free survival (*n* = 4929), DMFS: distant metastasis-free survival (*n* = 2765). (**G**) Petal plot of genes related to INHBA expression in breast cancer analyzed in TCGA database. (**H**) Volcano plot of differentially expressed genes COL10A1, COL11A1 and COL4A1 between CAFs with low and high expression of INHBA based on analysis of CAF RNA-seq data. (**I**) Correlation between INHBA expression and infiltration of CD8+ T cells, CD4+ T cells and B cells in BRCA analyzed by TIMER2.0. Horizontal coordinate values indicate proportion of immune cell infiltration. (**J**,**K**) Correlation between INHBA expression and infiltration of CAFs in BRCA, Basal, HER2+, Luminal A and Luminal B analyzed by TIMER2.0. Horizontal coordinate values indicate TIDE scores, with larger values having a higher percentage of CAF infiltration.

The clinical relevance of INHBA expression with breast cancer was analyzed by using the TCGA database. Results showed that INHBA gene expression was significantly up-regulated in breast tumor tissues compared to normal breast tissues (Figure 6A), and the same results were observed across all pathological types of breast cancer, including Basal, HER2+, Luminal-A, and Luminal-B types (Figure 6B). However, the expression of INHBA was not significantly correlated with stages of breast cancer (Figure 6C), tumor mutational burden (TMB) (Figure 6D), and microsatellite instability (MSI) (Figure 6E). In addition, we analyzed the correlation between INHBA expression and breast cancer prognosis by Kaplan–Meier, and results showed that patients with high INHBA expression had short overall survival (OS), recurrence-free survival (RFS), and distant metastasis-free survival (DMFS) (Figure 6F).

We subsequently assessed the set of INHBA-related genes in breast cancer and found that genes encoding collagens COL10A1, COL12A1, COL5A2 and COL11A1 were associated with INHBA expression (Figure 6G). More interestingly, the DEGs between CAFs^reduce^ and CAFs^not^ that we obtain through RNA-Seq analysis were significantly enriched in the extracellular matrix pathways (Figure 2C), and among them, COL10A1, COL11A1 and COL4A1 were significantly up-regulated in CAFs^reduce^ compared to CAFs^not^ (Figure 6H). This suggests to us that INHBA expression may be associated with cell infiltration.

Subsequently, TIMER2.0 analysis confirmed this speculation. Results showed that INHBA expression was significantly negatively correlated with CD8+ T cells and B cell infiltration, but it was not associated with CD4+T cells (Figure 6I). Meanwhile, INHBA expression was significantly positively correlated with the level of CAF infiltration (Figure 6J).

## 4. Discussion

In this study, we systematically elucidated the key role of CAFs in breast cancer treatment resistance and their molecular mechanisms. CAFs, main stromal cells in tumor microenvironment, are always in dynamic crosstalk with tumor cells and nourish the tumor to promote tumor progression [11,30]. For experimental model selection, the selection of MCF7 (low-invasive) and MDA-MB-231 (high-invasive) cells was based on their well-established representation of distinct invasive behaviors, widespread use in breast cancer research, and standardized experimental protocols. Although these models do not encompass all breast cancer subtypes, they remain fundamental for investigating low- versus high-invasiveness phenotypes in basic research. It has been well established in previous studies that CAFs are capable of inducing chemotherapeutic drug resistance in tumor cells [31]. In our experiments, we found that although the proteasome inhibitor, ixazomib, showed significant toxic effects on both breast cancer cell lines and CAFs, there were significant differences in the effects of CAFs from different patient sources on drug sensitivity. Specifically, CAFs^reduce^ were able to reduce the sensitivity of MCF7 cells to ixazomib, whereas CAFs^not^ did not exhibit this effect. Notably, in the MDA-MB-231 cell line, none of the CAFs tested showed a significant effect on the therapeutic efficacy of ixazomib. This finding suggests that not all CAFs from different individuals have identical effects on cancer cells, regardless of whether these cancer cells have the same pathological characteristics, reminding us that when CAF is considered one of the chemotherapy targets for breast cancer treatment, the pathological characteristics of CAFs in each patient should be considered.

Recent studies have shown that different subpopulations of CAFs are involved in the process of tumor drug resistance through multiple mechanisms [32]. The results of our transcriptome analyses suggest that such functional differences in CAFs may be mainly related to the expression levels of their INHBA genes. Differentially expressed gene (DEG) analysis further revealed that the gene expression differences between CAFs^reduce^ and CAFs^not^ were significantly enriched in the TGF-β signaling pathway and extracellular microenvironment-related pathways. This finding is consistent with previous findings showing that INHBA can participate in malignant tumor progression by regulating cancer cell signaling [18,33]. Through in-depth analysis of single-cell sequencing data from breast cancer tissues, we found that there were significant individual differences in the expression levels of INHBA in CAFs from different patient sources. A previous review suggested that INHBA might serve as a therapeutic marker [34], and the data in this study provide a further experimental basis for its expanded application in more types of cancer than multiple myeloma.

We demonstrated that Activin A secreted by CAFs reduced the sensitivity of MCF7 to ixazomib by in vitro and in vivo experiments. Considering that the sensitivity of proteasome inhibitors is related to proteasome activity [25,26], we investigated the effect of Activin A on proteasome activity and explored the mechanism, and declared that CAFs with high secreted levels of Activin A may reduce the sensitivity of breast cancer cells to ixazomib, which might involve the ERK1/2 signaling pathway-mediated inhibition of proteasome activity. Our study suggests that Activin A suppresses p-ERK in MCF7 cells while an existing study [35] reports that Activin A activates p-ERK. It seems contradictory. Actually, Activin A activates a variety of signaling pathways including p38-MAPK, ERK1/2, JNKs, and PI3K-AKT, but its mode of action is significantly cell-type-specific. For example, in neuronal PC12 cells, Activin A regulates autophagy by inhibiting JNK and p38-MAPK pathways [36]; in cerebral ischemia models, it reduces neuronal apoptosis by inhibiting JNK/p38-MAPK [37]; in K562 cells, it regulates autophagy by inhibiting ERK pathway and activating PI3K-AKT [38]. The integration of ours and other studies suggests that the signaling mechanism of Activin A is highly dependent on cell type. To the best of our knowledge, it is the first report that CAF-secreted Activin A has the ability to reduce the sensitivity of cancer cells to ixazomib; the presence of FST reversed this phenomenon, suggesting that targeting Activin A secreted by CAFs is a therapeutic target with which to overcome ixazomib resistance in MCF7 cells, or that ixazomib may be used to treat breast cancer patients with low expression levels of INHBA in CAFs.

Our finding that the inhibition of ERK phosphorylation levels by Activin A led to a reduction in proteasome activity is similar to a previous study in which a decrease in ERK phosphorylation levels in MCF7 led to a reduction in proteasome viability [39]. Here, we pondered the reason for this phenomenon, and we hypothesized that the reduced proteasome viability is related to the transcription of proteasome subunit components. It has been in the literature that the expression of 26S proteasome components is largely regulated by the antioxidant response element (ARE) [40], of which the ARE-related proteins, nuclear respiratory factor 1 (NRF1) and nuclear respiratory factor 2 (NRF2), are two of the most important transcription factors [41]. It was also reported that NRF1 and NRF2 are associated with the ERK pathway [42,43], which will be an important direction for our subsequent further studies.

In addition, we found that high expression of INHBA in breast tumor tissue was associated with the reduced survival of patients. INHBA expression in breast cancer tissues is closely associated with collagen production [44]. This, in turn, is an important part of ECM remodeling and leads to an immunosuppressive microenvironment [45]. This study analyzed the TCGA database and found that FAP and INHBA were significantly positively correlated with breast cancer. The literature indicates that FAP participates in ECM remodeling [46], while INHBA+CAFs also show ECM pathway enrichment. This suggests that FAP and INHBA may synergistically regulate ECM remodeling, which will be a focus of future research. Other studies have showed that immune cell infiltration is associated with a good prognosis [47], whereas the infiltration of CAF is associated with a poor prognosis [48]. Interestingly, we found the expression of INHBA was negatively correlated with the level of infiltration of CD8+ T and B cells, and was significantly positively correlated with the infiltration of CAFs cells. This is similar to the findings in a previous report in which subsets of CAFs with high expression of INHBA lead to the immunosuppressive microenvironment that prevents the infiltration of CD8+T cells in ovarian cancer [49]. These findings suggest that INHBA expression is associated with poor prognosis in breast cancer patients and, and INHBA plays an important role in regulating CD8+T cell, B cell, and CAF cell infiltration in the breast cancer tumor microenvironment.

## 5. Conclusions

Our study demonstrates that ixazomib exerts significant cytotoxic effects on both MDA-MB-231 and MCF7 breast cancer cell lines. However, in the presence of CAFs within the tumor microenvironment, ixazomib’s cytotoxicity displays subtype specificity. Specifically, CAFs with high INHBA expression can attenuate MCF7 cell sensitivity to ixazomib through Activin A secretion, which may be related to the down-regulation of ERK1/2 signaling pathway-mediated inhibition of proteasome activity. The limitation of this study is that our conclusions are based on in vivo and in vitro experiments, and further clinical trials are needed to support the extended use of ixazomib in the clinic, except in cases of multiple myeloma.

## Data Availability

The original contributions presented in this study are included in the article/Supplementary Materials. Further inquiries can be directed to the corresponding author.

## Supplementary Materials

The following supporting information can be downloaded at: https://www.mdpi.com/article/10.3390/biom15091318/s1, Figure S1: Visualization of the GSE176078 single-cell dataset. (A) Single-cell sequencing analysis UMAP visualization and clustering, with cell populations labeled by their major lineages, including T cells, epithelial cells, myeloid cells, endothelial cells, CAFs and B cells. (B) UMAP plot depicting INHBA expression levels for all cell types. (C) UMAP plot depicting CAF subtypes. (D) INHBA expression levels of CAFs subtypes.; Figure S2: Differential expression of INHBA in CAF of 26 breast cancer patients from GSE176078 dataset.; Figure S3: The full uncropped Blots for Figure 4B,C; Table S1: Clinical parameters of the four breast cancer patients.

## Abbreviations

The following abbreviations are used in this manuscript:

BC	breast cancer
CAFs	cancer-associated fibroblasts
FST	follistatin
PBS	phosphate-buffer solution
RNA-Seq	RNA-sequence
CM	conditioned medium
CCK-8	cell counting kit-8
RT-qPCR	real-time quantitative PCR
RFU	relative fluorescence unit
DEG	differential gene expression
KEGG	kyoto encyclopedia of genes and genomes
GO	gene ontology
GSEA	gene set enrichment analysis
ECM	extracellular matrix
scRNA-Seq	single-cell RNA sequencing
TMB	tumor mutational burden
MSI	microsatellite instability
OS	overall survival
RFS	recurrence-free survival
DMFS	distant metastasis-free survival
ARE	antioxidant response element
NRF	nuclear respiratory factor
