# Supplementary material for: Activin A Secreted by Cancer-Associated Fibroblasts Reduces the Sensitivity of Breast Cancer Cells to Ixazomib via Inhibition of Proteasome Activity
Source: Biomolecules. 2025 Sep 15;15(9):1318. doi: 10.3390/biom15091318 (PMC12466986; doi:10.3390/biom15091318)
Supplement: Supplementary file 1 [file biomolecules-15-01318-s001.zip › biomolecules-3687182-supplementary.pdf]

Supplementary Materials

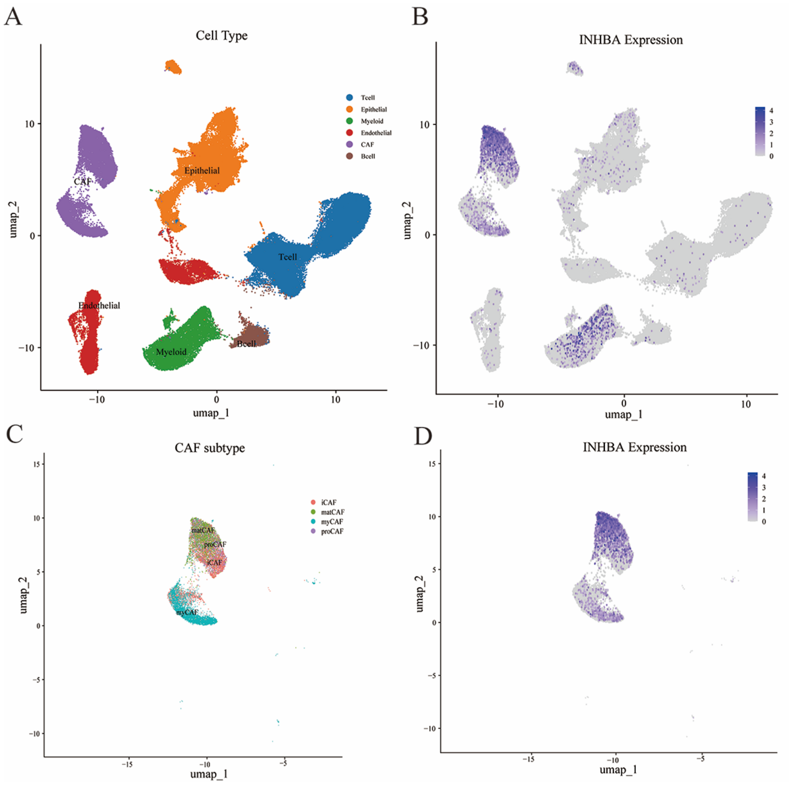

Figure S1. Visualization of the GSE176078 single cell dataset. (**A**) Single-cell sequencing analysis UMAP visualization and clustering, with cell populations labelled by their major lineages, including T cells, epithelial cells, myeloid cells, endothelial cells, CAFs and B cells. (**B**) UMAP plot depicting INHBA expression levels for all cell types. (**C**) UMAP plot depicting CAF subtypes. (**D**) INHBA expression levels of CAFs subtypes.

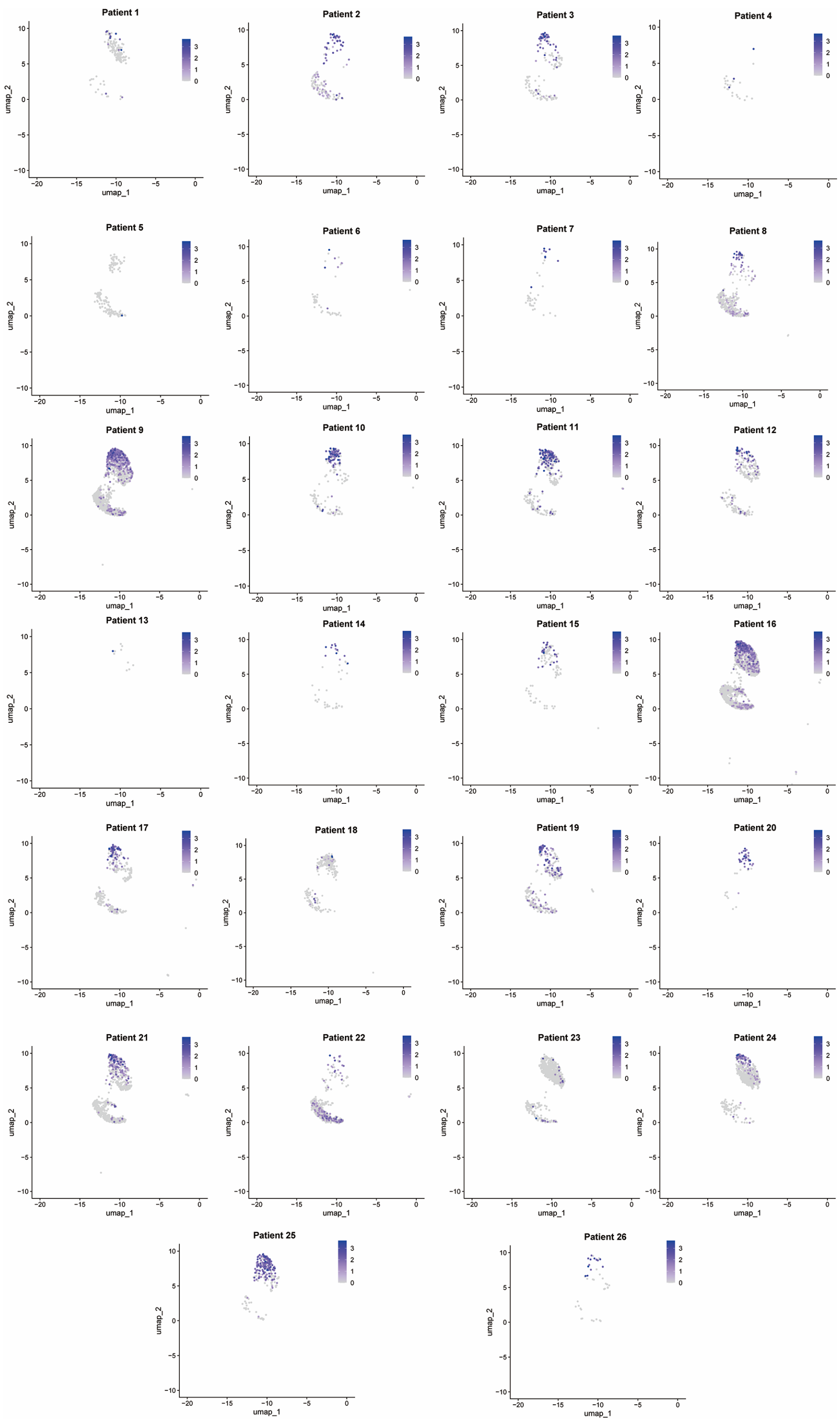

Figure S2. Differential expression of INHBA in CAF of 26 breast cancer patients from GSE176078 dataset.

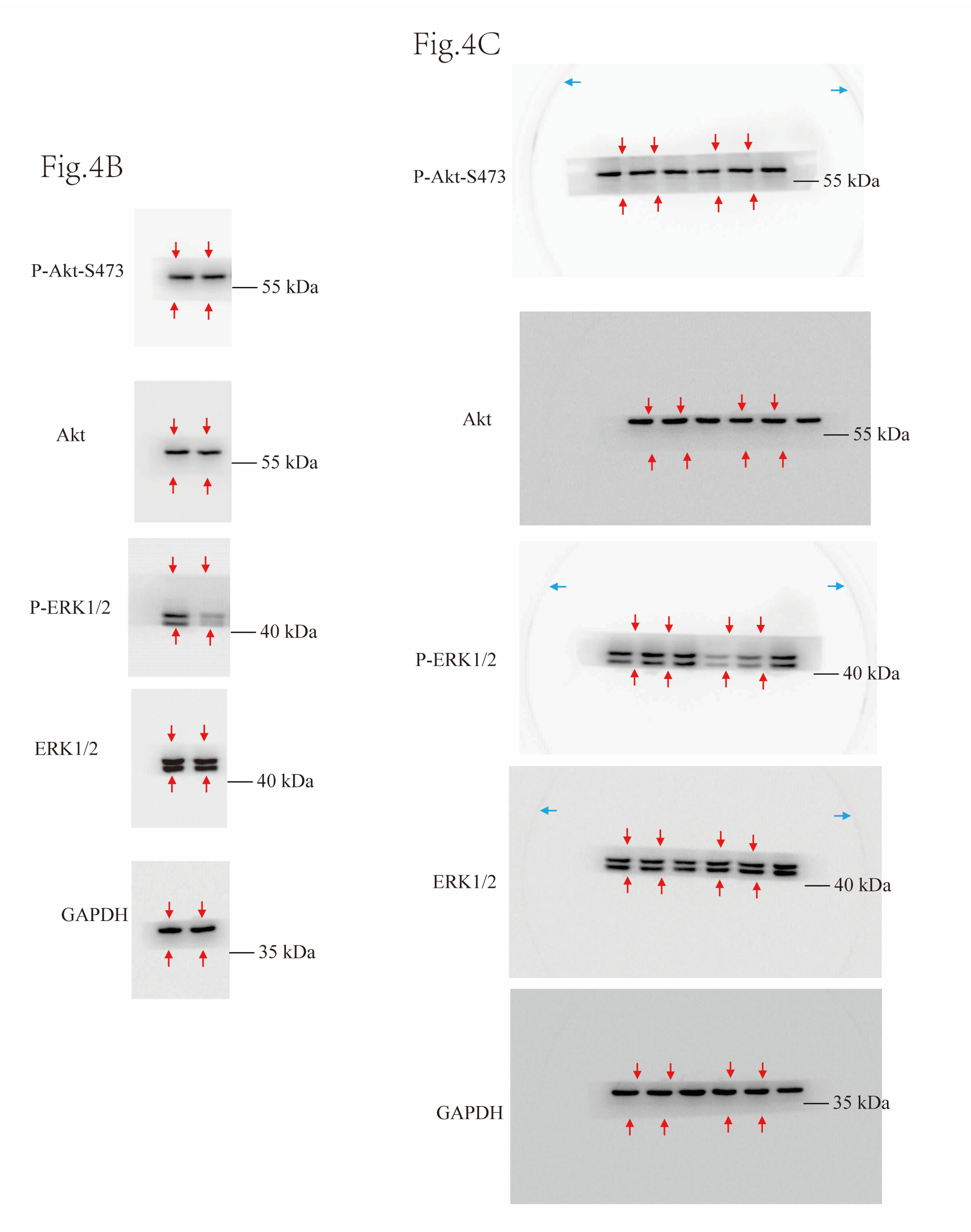

Figure S3. The full uncropped Blots for Figure4B and 4C. (In the figure, red arrows indicate the PVDF membrane boundaries, and blue arrows mark the development tank edges. It should be specifically noted that, to conserve antibody usage, the PVDF membrane was cut after transfer to retain only the regions containing target bands. In the development results, the membrane edges were clearly visible within the tank, and all images are original data without any post-processing.)

Table S1. Clinical parameters of the four breast cancer patients.

Patients	Histology	Her2	ER	PR	Ki67
**1**	IBC-NST	1+	80%+	60%+	20%+
**2**	IBC-NST	1+	80%+	70%+	10%+
**10**	IBC-NST	2+	85%+	80%+	40%+
**14**	IBC-NST	2+	90%+	80%+	10%+
